# Umbelliferone alleviates hepatic injury in diabetic db/db mice via inhibiting inflammatory response and activating Nrf2-mediated antioxidant

**DOI:** 10.1042/BSR20180444

**Published:** 2018-08-29

**Authors:** Jiangning Yin, Hanqing Wang, Guoyuan Lu

**Affiliations:** 1 Nephrology Department, the First Affiliated Hospital of Soochow University, No. 188 Shizi Road, Suzhou, Jiangsu, 215006, China; 2 Emergency Department, Affiliated Hospital of Jiangsu University, No. 438 Jiefang Road, Zhenjiang, Jiangsu 212001, China; 3 College of Pharmacy, Ningxia Medical University, No. 1160 Shengli Road, Yinchuan, Ningxia 750004, China

**Keywords:** HMGB1/TLR4/NF-κB, Inflammation, liver injury, Nrf2, Umbelliferon

## Abstract

The current study was designed to investigate the protective effect and possible mechanisms of umbelliferone (Umb) on liver injury in diabetic C57BL/KsJ-db/db (dbdb) mice. Mice were divided into five groups: wild-type mice group (WY), dbdb mice group, dbdb mice + Metformin (100 mg/kg) group, dbdb mice + Umb (20, 40 mg/kg) group. Blood glucose regulation was assessed by an oral glucose tolerance test (OGTT). At 28 days after drug administration, blood samples were obtained for the analysis of lipids and enzymes related to hepatic function, including alanine aminotransferase (ALT), aspartate aminotransaminase (AST) and total cholesterol (TC) and triglyceride (TG). Expression levels of inflammatory cytokines (TNF-α, IL-1β, and IL-6) and oxidative stress indicators (SOD and MDA) were measured with ELISA kit. The expressions of high-mobility group box 1 (HMGB1), Toll-like receptor (TLR) 4 (TLR4), Myd88, NF-κB, IκB, Nrf2, and HO-1 proteins were also evaluated by Western blotting analysis. The results showed that Umb significantly restored the blood glucose in OGTT, and inhibited the levels of insulin, TG, TC, as well as activities of ALT and AST. Moreover, Umb inhibited diabetic inflammation through down-regulating the expression of HMGB1, TLR4, NF-κB, and IκB. In addition, Umb alleviated oxidative damage in the liver by activating Nrf2-mediated signal pathway. These findings demonstrated that Umb exhibited protective effect against diabetic live injury, which may be through inhibiting HMGB1-induced inflammatory response and activating Nrf2-mediated antioxidant.

## Introduction

Diabetes mellitus, a major global public health challenge, is characterized by chronic hyperglycemia resulting from disturbances in insulin secretion, insulin action, or both [1]. Global number of people with diabetes in 2013 was 382 million, and this number is estimated to reach 592 million by 2035 [2]. Diabetes mellitus causes abnormalities in carbohydrate, fat, and protein metabolism, which may lead to serious complications such as blindness, renal failure, liver injury, nerve damage, and atherosclerosis [3,4]. Amongst the complications of diabetes, liver injury is a major problem and has been gradually paid much attention [5,6]. Diabetes-induced liver injury is closely related to inflammatory responses, liver fibrosis, and lipid accumulation [7]. Amongst these, liver inflammation is a critical mechanism and anti-inflammatory agents have been reported to protect against diabetes-induced liver injury [8]. Therefore, anti-inflammation may be a possible effective therapeutic method for this disease.

High-mobility group box 1 (HMGB1), a highly conserved nuclear protein, affects a variety of biological processes including inflammatory diseases. In addition to its nuclear roles, HMGB1 in the extracellular fluid also acts as a damage-associated molecular pattern molecule (DAMP) in processes such as inflammation and cell migration [5]. HMGB1 participates in inflammation, where it acts as a proinflammatory cytokine [9]. Under inflammatory conditions, HMGB1 is passively released or actively secreted from affected monocytes/macrophages into the extracellular environment [9]. Consequently, HMGB1 combines with receptor for advanced glycation end products or Toll-like receptors (TLRs). Finally, nuclear factor (NF)-κB could be activated and the inflammatory response mediated [10]. Recently, HMGB1 has been found to converge a spectrum of pathophysiological signals triggered by the diabetic milieu, and has been reported as promising molecular targets for treatment in diabetes [11].

Umbelliferone (Umb) or 7-hydroxycoumarin, a derivative of coumarin, is abundant in many plants such as carrot, coriander, and garden angelica. Umb has displayed a wide range of pharmacological activities. It is reported to have antioxidant, anti-inflammatory, antidiabetic and antitumor activities [12]. These biological activities of Umb make it a good candidate to be studied in the treatment of diabetic liver injury. To our knowledge, no previous work has been investigated whether Umb had a therapeutic effect on diabetic liver injury in C57BL/KsJ-db/db (dbdb) mice. Therefore, in the present study, we aim to determine whether Umb attenuates diabetes-induced liver injury, and elucidate the underlying mechanisms. Our findings reveal novel metabolic activities of Umb in the liver, which point to the potential use of Umb in the treatment of hepatic injury associated with diabetes mellitus.

## Materials and methods

### Reagents

Umb (98%) was purchased from the National Institute for the Control of Pharmaceutical and Biological Products (Beijing, China). Alanine aminotransferase (ALT), aspartate aminotransaminase (AST), total cholesterol (TC), and triglyceride (TG) commercial kits were purchased from Nanjing Jiancheng Bioengineering Institute (Nanjing, China). ELISA kits for determination of insulin, IL-1β, IL-6, and TNF-α were provided by Nanjing KeyGEN Biotech. Co., Ltd. (Nanjing, China). All the antibodies were provided by Cell Signaling Technology (Danvers, U.S.A.).

### Animals

 dbdb mice were purchased from Model Animal Research Center of Nanjing University (MARC, Nanjing, China) and housed at 23 ± 2°C with a 12-h light/dark cycle. Water and food were provided *ad libitum*. All the experimental procedures were performed strictly according to the National Institutes of Health Guidelines for the Care and Use of Laboratory Animals and approved by Wenzhou Medical University.

### Experimental design

After 1 week of feeding adaptation, all the mice were divided into five groups (*n*=12): Group 1 (wild-type mice group, WY): wild-type C57BLKS mice were orally treated with saline. Group 2 (db/db control): db/db mice were treated with normal saline orally. Group 3 (Metformin): db/db mice were treated intragastrically with metformin at a dose of 100 mg/kg/day. Group 4 (Umb-20): db/db mice were treated intragastrically with Umb at a dose of 20 mg/kg/day. Group 5 (Umb-40): db/db mice were treated intragastrically with Umb at a dose of 40 mg/kg/day. All doses were given for 4 weeks. At the end of experiment, blood samples were collected from carotid artery and centrifuged. The supernatants were stored at −80°C for biochemical analysis. Additionally, liver tissues were removed, rinsed with a physiological saline solution, and immediately stored at −80°C for Western blotting analysis.

### Oral glucose tolerance test

At the 29th day post treatment, oral glucose tolerance tests (OGTT) were performed whereby overnight fasted mice received glucose (2 g/kg) via gavage feeding at 08:00 a.m. Blood samples were collected from the tail vein at 0, 30, 60, 90, and 120 min after glucose treatment. Glucose concentrations were evaluated by using a glucose analyzer (SureStep, Lifescan, Inc., Milpitas, CA).

### Biochemical estimations

Serum levels of ALT, AST, TC, and TG in animal serum were determined using commercial kits (Nanjing Jiancheng Bioengineering Institute, Nanjing, China). The fasting insulin was measured after fasting for 12 h at the end of the experiment. The concentration of insulin in the serum was measured with insulin ELISA kit (R&D Systems, Abingdon, U.K.). All commercial kits were used following manufacturer’s instructions.

### Determination of TNF-α, IL-1, and IL-6 in serum and liver tissues

Inflammatory cytokines, including IL-6, IL-1β, and TNF-α levels in the serum and liver tissue, were measured using ELISA kits according to the manufacturer’s instructions. The optical density (OD) of each well was read at 450 nm, and the concentrations of inflammatory cytokines were quantitated by reference to the standard curves.

### Determination of SOD and MDA in serum and liver tissues

Malonaldehyde (MDA) and superoxide dismutase (SOD) assay kits were purchased from Nanjing Jiancheng Bioengineering Institute (Nanjing, China). Levels of MDA and activity of SOD were measured by following the commercial kits’ protocols.

### Histological examination

The liver tissues were carefully removed, formalin-fixed, and embedded in paraffin. Then, the samples were sectioned into 3-μm-thick slices, and stained with Hematoxylin-Eosin (Nanjing Jiancheng Bioengineering Institute, Nanjing, China). The pathological changes in the liver tissues were examined using a light microscope (Olympus Medical Systems Corp., Tokyo, Japan), and the degree of lipid infiltration was scored on a scale of 0–4, with 0 being normal healthy tissue and 4 being the worst, as described previously [14].

### Western blot

The liver tissues were homogenized, washed with PBS, and lysed in a commercial RIPA buffer (Beyotime, Nanjing, China). After centrifugation at 12000 rpm for 20 min, the dissolved proteins were obtained from the supernatant. The protein concentrations were quantitated using a BCA protein assay (Beyotime, Nanjing, China). Equal amounts of proteins were loaded by SDS/PAGE (10% gel) and were electrotransferred to nitrocellulose membranes. After the membranes were blocked, they were incubated with primary antibodies against Nrf2, HO-1, HMGB1, TLR4, P65, p-P65, IκB, p-IκB, or GAPDH overnight at 4°C, followed by horseradish peroxidase-conjugated second antibodies for 2 h at room temperature. The protein bands were visualized using an ECL detection reagents and a gel imaging system (Tanon Science & Technology Co., Ltd., China). Normalization of total protein expression was carried out using GAPDH as control.

### Statistical analysis

Data are expressed as means ± S.D. of at least three separate experiments. One-way ANOVA, followed by Tukey’s multiple comparison test were used to determine statistical differences. A *P*-value of less than 0.05 was considered statistically significant.

## Results

### Effect of Umb on OGTT

**Figure 1:**
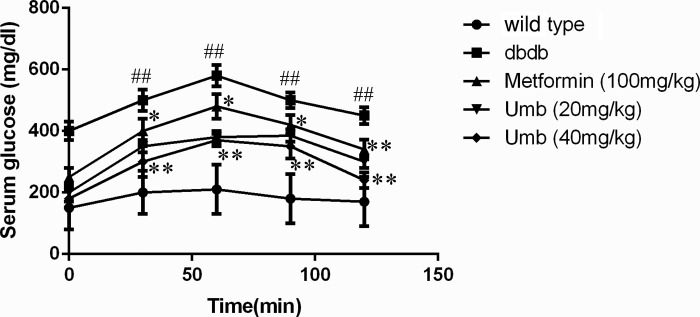
Effects of Umb on OGTT The data are expressed as mean values ± S.D. ^##^*P*<0.01 compared with wild-type group. **P<0.01, *P<0.05 compared with dbdb group.

From the curve of OGTT (Figure 1), it was shown that the levels of blood glucose in the dbdb mice group were distinctly higher than those in the wild-type mice group. After treatment with Umb (20, 40 mg/kg), blood glucose in db/db mice were significantly decreased as compared with db/db mice treated with saline. Similarly, metformin (100 mg/kg) reduced blood glucose concentrations after the oral glucose administration.

### Effect of Umb on hepatic pathological changes

**Figure 2:**
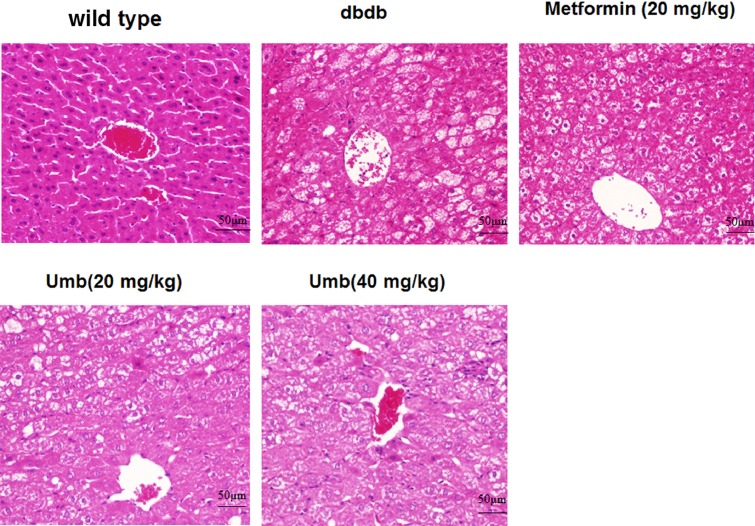
Effects of Umb on histological examination (×200)

Morphological changes of the liver are shown in Figure 2. As presented in the Figure 2, lipid accumulation was observed in liver tissues of the dbdb mice. Lipid vacuoles occupied much of the hepatocyte cytoplasm, pushing the nucleus to the periphery of the cell. Additionally, some hepatocytes appeared bloated. Umb (20, 40 mg/kg) and metformin (100 mg/kg) treatments strikingly attenuated the extent of steatosis. These lipid droplets were noticeably reduced both in size and number in the liver of dbdb mice treated with Umb (20, 40 mg/kg) and metformin (100 mg/kg), suggesting that these treatment effectively inhibits lipid accumulation in liver.

### Effect of Umb on TG, TC, insulin, AST, and ALT in serum

**Figure 3:**
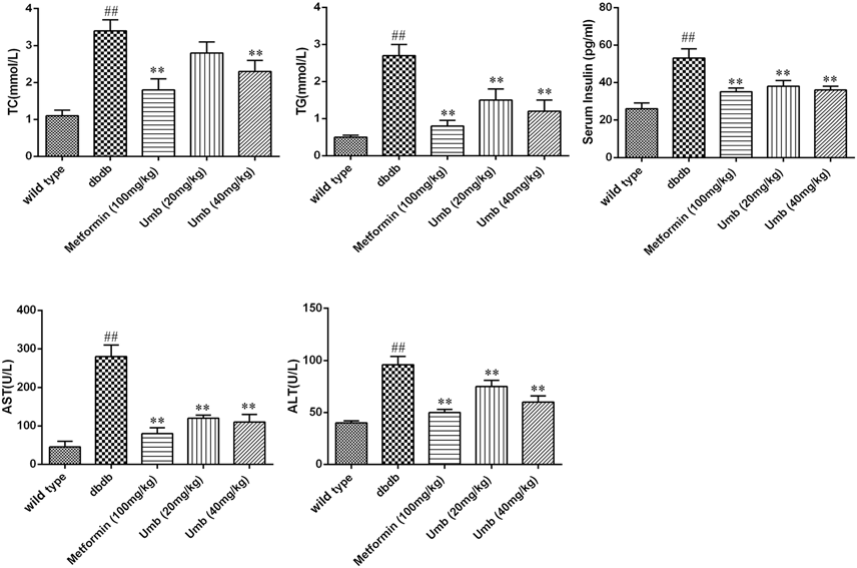
Effect of Umb on TC, TG, insulin, AST, and ALT in serum The data are expressed as mean values ± S.D. ^##^*P*<0.01 compared with wild-type group. **P<0.01 compared with dbdb group.

As illustrated in Figure 3, the serum levels of TG and TC were significantly higher in db/db mice than in the normal control group (*P*<0.01). However, Umb (20, 40 mg/kg) treatment remarkably reduced TG and TC levels in a dose-dependent manner. To investigate the effect of Umb on hepatic function, serum level of insulin, AST, and ALT activities were determined. As shown in Figure 3, the serum levels of insulin, AST, and ALT the db/db mice group were significantly higher than those of the control group, and Umb (20, 40 mg/kg) and metformin (100 mg/kg) treatment significantly reduced the serum insulin, AST, and ALT as compared with the model group.

### Effect of Umb on levels of TNF-α, IL-6, and IL-1β in serum and liver

**Figure 4:**
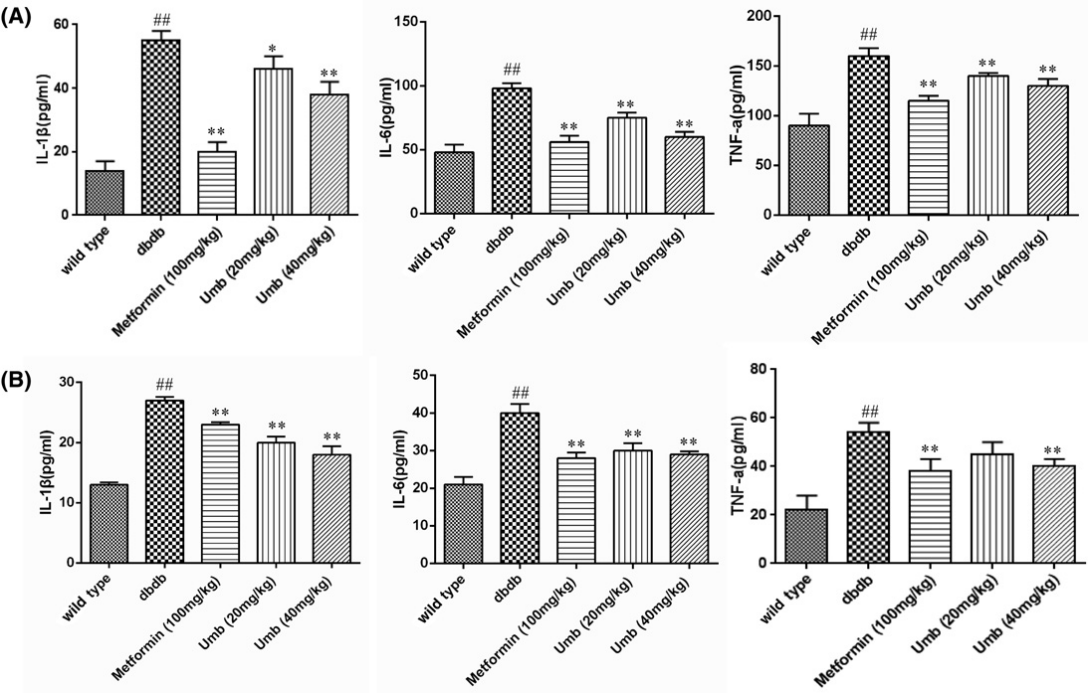
Inflammatory cytokines levels in the serum and liver tissue Effect of Umb on TNF-α, IL-6, and IL-1β in serum (**A**) and liver (**B**). The data are expressed as mean values ± S.D. ^##^*P*<0.01 compared with wild-type group. **P<0.01, *P<0.05 compared with dbdb group.

Inflammation is associated with hepatic steatosis in diabetic liver injury. To explore the effects of Umb on inflammation, the levels of TNF-α, IL-6, and IL-1β were analyzed in the serum and liver tissue of db/db mice. It was observed that the serum and hepatic TNF-α, IL-6, and IL-1β content of db/db mice group were significantly higher than those of the normal control group (*P*<0.01). However, Umb treatment notably decreased the levels of TNF-α, IL-1β, and IL-6 in serum and liver tissue in a dose-dependent manner when compared with the db/db control group (Figure 4).

### Effect of Umb on HMGB1/TLR4/NF-κB signaling pathway

**Figure 5:**
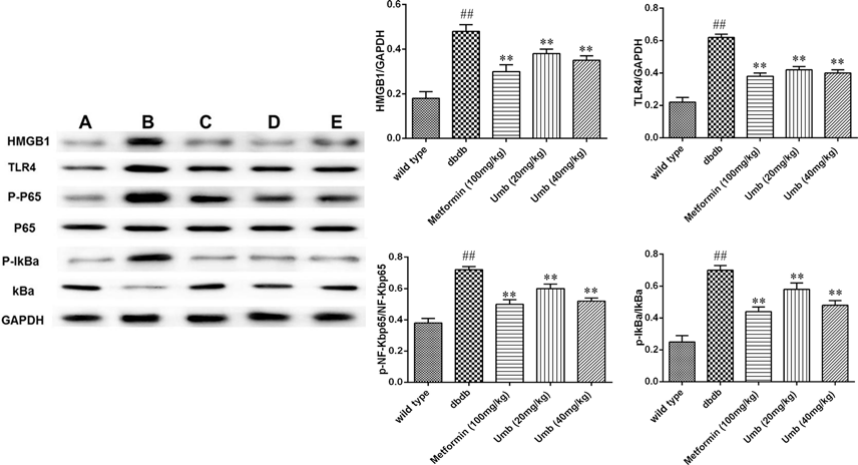
Effects of Umb on HMGB1/TLR4/NF-κB signaling pathway in liver tissues of diabetic db/db mice A: Wild-type, B: dbdb, C: Metformin (100 mg/kg), D: Umb (20 mg/kg), E: Umb (40 mg/kg). The data are expressed as mean values ± S.D. ^##^*P*<0.01 compared with wild-type group. **P<0.01 compared with db/db group.

**Figure 6:**
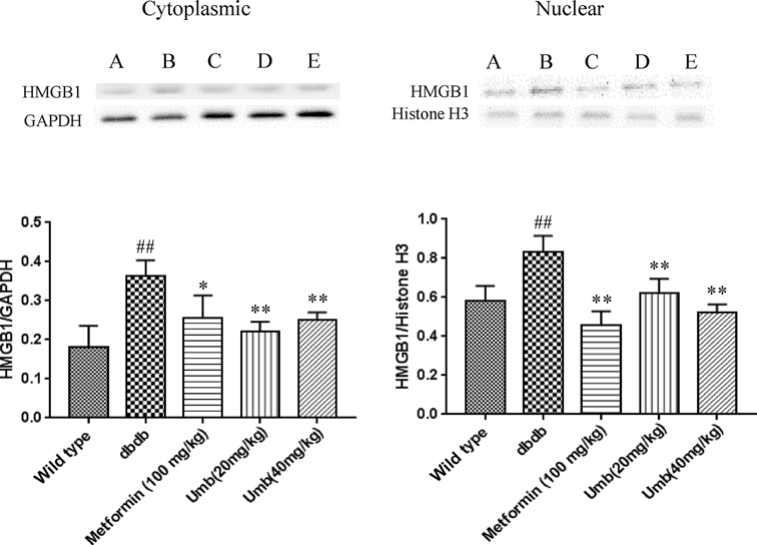
Effects of Umb on cytoplasmic and nuclear HMGB1 protein expressions The cytoplasmic and nuclear protein was extracted separately and HMGB1 was detected by Western blot. ^##^*P*<0.01 compared with wild-type group. **P<0.01, *P<0.05 compared with db/db group.

HMGB1/TLR4/NF-κB signaling pathway participates in regulating the inflammatory cascades [13]. To investigate whether HMGB1/TLR4/NF-κB pathway might be responsible for the effects of Umb on liver inflammation in db/db mice, Western blot experiments to analyze the expression levels of HMGB1, TLR4, Myd88, NF-κB, and IκB and their phosphorylation. As shown in Figure 5, it was found that the expressions of HMGB1, TLR4, p-NF-κBP65, and p-IκB were significantly up-regulated in liver tissues of dbdb mice. HMGB1 cytoplasmic and nuclear proteins also increased in the db/db mice, compared with that of wild-type mice (*P*<0.01) (Figure 6). Treatment with Umb (20, 40 mg/kg) and metformin (100 mg/kg) obviously ameliorated these changes. The obtained results demonstrated that Umb could successfully block the expressions of HMGB1/TLR4/NF-κB pathway.

### Effect of Umb on diabetic oxidative stress

**Figure 7:**
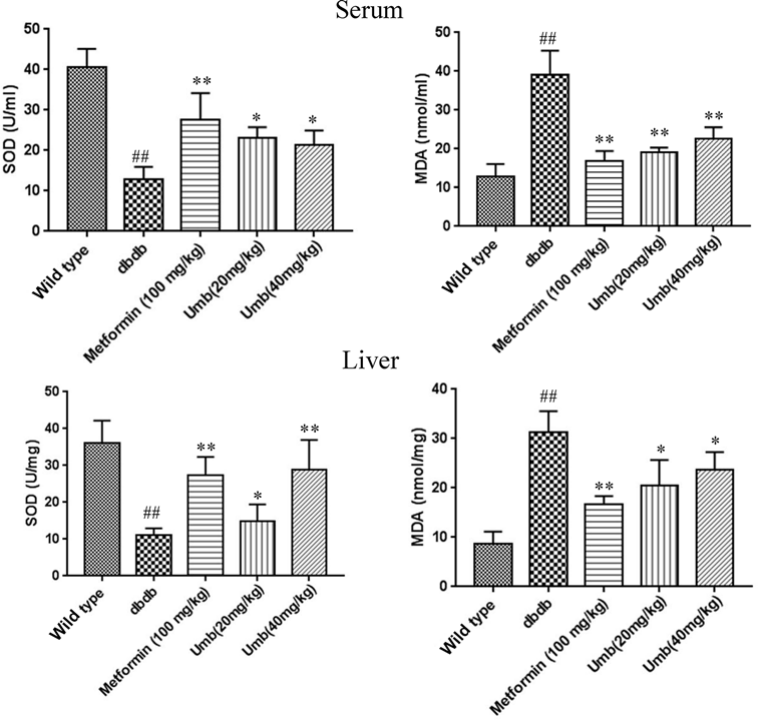
Effect of Umb on SOD and MDA levels in serum and liver ^##^*P*<0.01 compared with wild-type group. **P<0.01, *P<0.05 compared with dbdb group.

It is well known that diabetes leads to hepatic oxidative stress, which can promote the development of liver injury [14]. Umb was reported to possess antioxidant activity. In order to evaluate the effect of Umb on the diabetic oxidative stress, antioxidant enzyme activities and lipid peroxidation were examined. As shown in Figure 7, the activity of SOD was significantly decreased in the db/db mice. However, the decrease in the enzyme was restored by Umb treatment in a dose-dependent manner (*P*<0.01). In addition, serum and hepatic levels of MDA in db/db mice group were significantly higher than those in the control group. However, these elevated levels were significantly reduced by Umb treatment at a dose of 20 and 40 mg/kg.

### Effect of Umb on Nrf2-mediated antioxidant response

**Figure 8.**
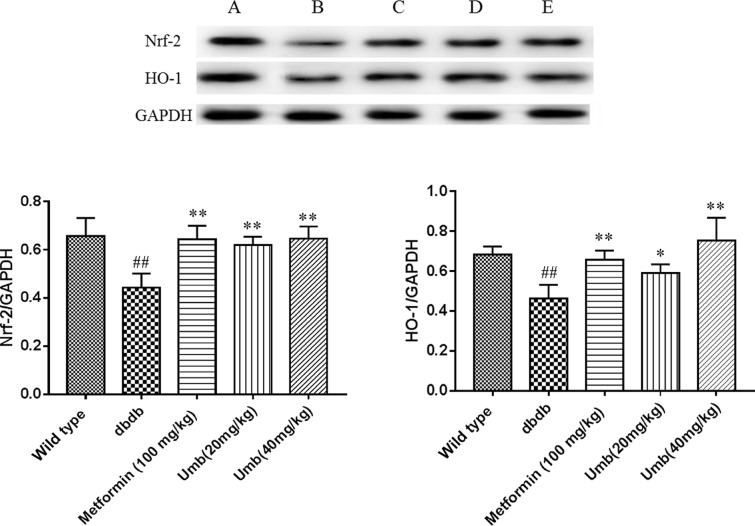
Effects of Umb on Nrf2 and HO-1 expressions in liver of db/db mice ^##^*P*<0.01 compared with wild-type group. **P<0.01, *P<0.05 compared with db/db group.

To explore the mechanism underlying the protective effects of Umb on oxidative damage, the expressions of Nrf2 and its downstream protein HO-1 were analyzed by Western blot analysis. As shown in Figure 8, Nrf2 and HO-1 in db/db mice group were remarkably down-regulated compared with the control group. However, Umb treatment significantly restored the expressions of these proteins compared with the db/db mice group. In general, these findings suggested that the hepatoprotective effect of Umb against hepatic injury in diabetic mice is associated with activation of the Nrf2-mediated antioxidant response.

## Discussion

Diabetes mellitus is characterized by hyperglycemia, a lackage of insulin action, insulin resistance, and the progression of diabetic pathology in the retina, renal glomerulus, and nerve [15]. Diabetes accelerated atherosclerotic diseases affecting arteries that supply the heart, brain, and lower extremities [16]. In addition, diabetic liver injury is also a very serious diabetic complication in our modern-day society. Diabetes and insulin resistance has been well identified as important factors in patients with diabetic liver injury [17–19]. In our present study, dbdb mice showed abnormal OGTT performance, lipid profiles and increased serum insulin, ALT, and AST levels. And the study indicated that Umb and metformin could improve liver function and shows hypoglycemic effect as reflected by the serum levels of bio-markers above-mentioned. These results were in agreement with the liver histological changes that were observed by H&E staining, confirming the effects of Umb on liver injury in the diabetic mice. Hence, Umb is a natural effectively hepatoprotective agent. In order to clarify how Umb protected against diabetic liver injury, we explored the possible molecular mechanism in the present study.

In the present study, we demonstrated that the levels of TNF-α, IL-1β, and IL-6 were elevated in diabetic mice compared with control groups, indicating that the liver inflammation existed in diabetic mice. Umb treatment significantly decreased hepatic TNF-a, IL-1β, and IL-6 levels in db/db mice. These inflammatory cytokines play critical roles in the progression of liver injury, provoking hepatocellular injury and death [20–22].TNF-α and IL-1β were sensitizing factors. They acted on leukocyte infiltration of the liver, and amplified hepatocyte damage [23,24]. IL-6 is a pro-inflammatory cytokine that has been proposed to have a direct and indirect role in induction inflammation [25]. Umb could attenuate these cytokines levels, indicating that the anti-inflammatory effect of Umb may partly contribute to the liver-protective effect of Umb in db/db mice.

In order to gain insight into the related molecular mechanisms of anti-inflammatory effect of Umb in db/db mice, we investigated inflammation-related HMGB1/TLR4/NF-κB pathway in the liver tissue of db/db mice. HMGB1 is a key mediator of inflammation during sterile- and infection-associated responses. It shares features with cytokines, and acts on the surface receptors of immune cells to induce expression of inflammatory factors and further HMGB1 release, resulting in promotion of the inflammatory cascade [26]. When hepatic failure occurs, HMGB1 can be constitutively expressed and passively released from damaged cells or actively secreted from activated immune cells. HMGB1 also can signal through TLR4, and then activate NF-κB and induce the expression of pro-inflammatory genes. These changes promote recruitment and activation of inflammatory cells, thereby resulting in the activation of inflammation [27]. The further Western blotting analysis presented that the levels of HMGB1, TLR4, NF-κB and IκB expression in the liver tissues of the diabetic dbdb mice were higher than in the wild type mice. Umb intervention obviously reversed these alterations, demonstrating Umb was responsible for the hepatoprotection due to its anti-inflammatory properties by reducing HMGB1/TLR4/NF-κB activation.

Oxidative stress also is a major cause of diabetic liver injury. The current study showed that Umb treatment significantly suppressed the levels of MDA, a marker of oxidative stress, in diabetic mice livers. It is well known that oxidative stress is normally counterbalanced by antioxidant defense systems [28]. The results of our study showed that diabetic condition significantly decreased the activities of SOD. However, Umb remarkably reversed the depletion of the antioxidant enzyme. Nrf2 is a critical transcription factor for encoding detoxification enzymes and antioxidant proteins to against oxidative stress [29]. Nrf2 can be activated by antioxidants, and binds to antioxidant-response elements (AREs) to activate the expressions of downstream genes including HO-1 and NQO1 [30]. In the present study, the results showed that Umb reversed the depletion of Nrf2 and its downstream target HO-1 in liver tissues of diabetic db/db mice. Taken together, the results indicate that Umb exhibits hepatoprotective effect against oxidative stress via activation of Nrf2 signal pathway.

Umb is a major and active coumarin compound, obtained from wide variety of dietary sources and natural medicinal plants. It has been shown to exert a wide range of pleiotropic therapeutic effects on diabetes and its complications, including the antihyperglycemic activity, the anti-inflammatory effect, and the inhibiting effect of oxidative-nitrosative stress, etc. [31–33]. The present study revealed the hepatoprotective effect of Umb, which was shown to decrease the HMGB1-related inflammatory responses and activating Nrf2-mediated antioxidant. The findings of the present study are limited and preliminary, in-depth mechanism study involving siRNA and gene knock-out technologies need to be performed to provide insight into how Umb regulate HMGB1 and TLR4 expression. However, the results of the present work open the possibility of new therapeutic perspectives of Umb for the treatment of hepatic injury in diabetes.

## Conclusion

In conclusion, the present study successfully investigated the protective effects of Umb in diabetic liver injury. The potential mechanism of Umb was related to the inhibition of the hepatic inflammation through HMGB1/TLR4/NF-κB pathway. The protective effects of Umb were also associated with reduction in oxidative stress via inhibiting oxidant production and up-regulating Nrf2-mediated antioxidant response. This work provides the first evidence in support of the use of Umb as potential therapeutic medicine for diabetic live injury.

## Abbreviations

ALT: alanine aminotransferase
AST: aspartate aminotransaminase
dbdb: C57BL/KsJ-db/db
HMGB1: high-mobility group box 1
HO-1: heme oxygenase-1
IκB: inhibitor of nuclear factor kappa-B
IL: interleukin
MDA: malondialdehyde
Myd88: myeloid differentiation primary response 88
NF-κB: nuclear factor κB
Nrf2: nuclear factor (erythroid-derived 2)-like 2
OGTT: oral glucose tolerance test
SOD: superoxide dismutase
TC: total cholesterol
TG: triglyceride
TLR: toll-like receptor
TNF-α: tumor necrosis factor α
Umb: umbelliferone
